# Integration of the fluorescence based portable device with the AI tools for the real-time monitoring of oral mucosal lesions

**DOI:** 10.1038/s41598-025-94676-w

**Published:** 2025-03-25

**Authors:** Pavan Kumar, Shashikant Rathod

**Affiliations:** 1 Faculty of Engineering and Technology (FEAT), Datta Meghe Institute of Higher Education and Research (DMIHER), Wardha, 442001 India; 2 Department of Physics, Indian Institute of Technology Kanpur (IITK), Kanpur, 208016 India; 3 Department of Instrumentation and Control Engineering, COEP Technological University, Pune, 411005 India; 4 Program Launch Management Analytics, FORDS Motors Pvt. Ltd, Chennai, 600119 India

**Keywords:** Oral mucosal lesions, Fluorescence spectroscopy, Porphyrin, AI tools, Health care, Medical research, Oncology, Optics and photonics, Physics

## Abstract

There is a need for non-invasive, sensitive, real-time, and user-friendly optical devices integrated with artificial intelligence (AI) based tools for the detection of oral mucosal lesions at early stage. Research on the development of optical devices has been executed by several research groups for the cancer detection and it is still being continued. We have also contributed towards it by developing a steady- state fluorescence-based portable device. The in-house developed device is equipped with 405 nm laser diode, UV visible spectrometer, optical components, and other accessories. Laser light irradiated on the oral cavity of diseased (cancerous) and non-diseased (normal) groups, excites the two endogenous fluorophores namely FAD and porphyrin. We observed an enhancement in the porphyrin fluorescence of cancerous patients (OSCC and Dysplasia) than the normal group. Data analysis carried out by AI tools i.e., Naïve Bayes, LDA, and QDA showed slightly higher accuracy for QDA. QDA was able to discriminate among Normal to OSCC, Normal to Dysplasia, and Dysplasia to OSCC with accuracies of 95.34%, 100%, and 97.43% respectively.

## Introduction

Tumor development is a slow process and it takes years before a tumor is felt by hand or can be visualized by the naked eye. It is therefore need of sensitive and non-invasive devices to identify the disease at the very early stage. Among the conventional methods, tissue biopsy with histopathology is the only standard screening procedure for the identification of oral lesions. Though, histopathology is the most reliable (gold standard) but its invasive ethos and time-consuming procedure make it incompetent. Patients at any stage of cancer i.e., benign, precancer (mild, moderate, or severe), and oral squamous cell carcinoma (OSCC; grade I to IV) have to undergo this painful process during the treatment. It can also not be assured that the selected area for the biopsy is rightly chosen or not. The research finding shows that a five-year survival rate after the treatment is extensively less (~ 45%), which has not remedied in the last few decades. The predominant reasons for poor survivability are late diagnosis as well as the absence of early symptoms^1–5^. It is therefore needed for such devices which to be sensitive, fast in detection, user-friendly, low cost, and portable.

Research carried out for the detection of oral lesions indicates that optical devices (spectroscopic and imaging) such as fluorescence, Raman, lifetime, diffuse reflectance, etc. could detect oral cavity abnormalities non-invasively as well as at an early stage^6–15^. Among the spectroscopic devices, steady-state fluorescence-based devices are adequately utilized by the researchers for in-vivo testing of oral cancer^16–30^. Fluorescence devices are based on light interaction with material (e.g. biomaterial, tissue, etc.). A biological tissue consists of several endogenous fluorophores and they have a particular excitation wavelength (λ_exc_) and emission wavelength (λ_emi_) at which they yield maximum emission and absorption bands. Tryptophan, Collagen, Elastin, NADH, FAD, and Porphyrin are the key fluorophores and they have excitation wavelengths near 280, 325, 350, 340, 450, and 400 nm and emission bands at 340, 390, 410, 440, 530, and 634 nm respectively. It has been established that fluorophore concentration changes with the progress of disease and these changes can be noticed in the fluorescence signal.

The utility of the fluorescence-based device for the detection of cancer (breast and lung) was first studied by the Alfano et. group in 1987 on human tissues. In this study, they observed the spectral differences between normal and malignant tissues^6^. Later on, several research groups carried out ex-vivo and in-vivo studies for the detection of cancers such as bladder, cervical, bronchial, lung, breast, etc. and noticed the differences in the spectral shapes & intensities. Here, we have summarized the key findings of the research carried out by the research groups for in-vivo detection of oral cancer using spectroscopic and imaging devices. Towards it, Gillenwater et al. conducted a clinical study on patients (*n* = 15) and healthy volunteers (*n* = 8) using a fluorescence spectroscopy system (λ_exc_ = 337 nm) and achieved a sensitivity of 88% and specificity of 100%^24^. Inaguma M and Hashimoto K group performed an in-vivo study on 78 oral carcinoma patients and reported the enhancement in porphyrin-like fluorescence on 66 carcinoma patients (85%)^25^. In a pilot study by Van Staveren HJ et al., group using the auto-fluorescence spectroscopy (λ = 420 nm) and ANN on leukoplakia patients (*n* = 21), noticed that leukoplakia could be demarcated to normal oral mucosa with 86% sensitivity and 100% specificity^26^. Further, Diana C.G. de Veld et al. group presented two detailed studies for in-vivo detection of oral cancer. They accomplished an anatomy-based study on 97 healthy volunteers only and noticed the variations in the fluorescence intensities among the anatomical sites as well as in another study they performed measurements on benign, dysplastic, and malignant lesions and distinguished the malignant lesions from healthy volunteers with ROC-AUC value of 0.97^11,27^. Majumder et al. group did a clinical study on 16 OSCC patients and 13 volunteers and reported that cancerous lesions could be demarcated to normal with 95% sensitivity and 96% specificity by the use of nonlinear MRDF on fluorescence data^28^. Jayanthi et al. group achieved AUC-ROC values of 0.98 for auto-fluorescence and 0.99 for DR spectroscopy^29^. Nazeer et al. group showed that fluorescence spectroscopy and multivariate analysis (PCA-LDA) could distinguish habitués, non-habitués, and leukoplakia with sensitivity values of 60 to 100% and specificity values of 76 to 100%^30^. Lane et al. group differentiated the lesions with sensitivity and specificity of 98 and 100% respectively by the fluorescence-based handheld device^31^. Pavlova et al. group conducted a study using a high-resolution fluorescence microscopy device on 49 oral biopsies and reported that auto-fluorescence properties vary significantly among the anatomic sites of oral cavity^32^. Roblyer et al. group performed measurements on 56 patients and 11 volunteers using an autofluorescence imaging system and achieved a sensitivity of 100% and specificity of 91% for the validation dataset^33^. Roblyer et al. group also utilized a multispectral digital microscope to improve the detection of oral neoplasia and observed an increased red fluorescence in cancerous lesions^34^. Our group also performed an in vivo study for oral cancer detection using an in-house developed fluorescence-based device. Classification done by machine learning methods like PCA, SVM, and ROC has shown excellent results^35^.

Here, we have presented in vivo detection of oral lesions by the use of in-house fabricated fluorescence device (steady-state) and carried out measurements on patients i.e., OSCC, dysplastic‚ and healthy volunteers. Major bands of FAD as well as major and minor bands of porphyrins were noticed in the fluorescence spectra of patients and volunteers. An advantage of our in-house spectroscopic device is its adaptability; by removing the focusing lens and replacing the spectrometer with a CCD, it can function as an imaging system, captures an area of 10 mm^2^. To distinguish among the groups, AI tools such as PCA, Naïve Bayes, LDA, QDA, ROC analysis were employed. To our knowledge, a detailed research among the classifiers for the discrimination oral lesions has yet to be reported.

## Materials and methods

### Instrumentation

**Fig. 1:**
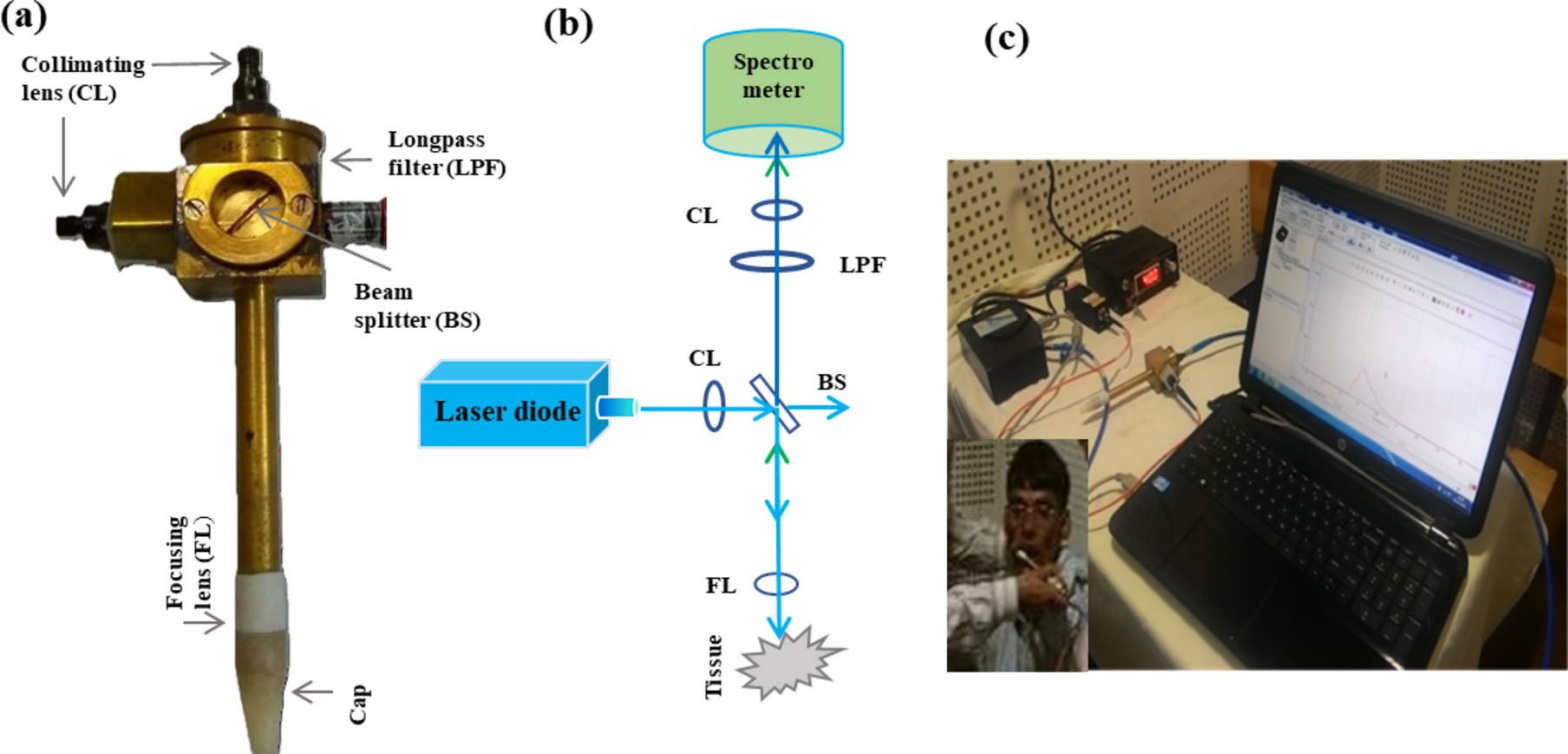
(**a**) Photograph of the fluorescence based in-house fabricated portable device (**b**) ray diagram (**c**) device photograph with all the needful accessories.

For in-vivo testing of patients and the control group, we developed a portable device based on fluorescence. To fabricate the portable device i.e., hardware parts, we utilized Brass and Teflon materials. The device was fabricated in the CELP workshop at IIT Kanpur, India. In the device, optical components i.e., collimating lens (CL), beam splitter (BS), long pass filter (LPF), and focusing lens (FL) were embedded in various compartments of the device as displayed in Fig. 1a. Apart from these components, other accessories such as a diode laser of 405 nm (Diode Laser 405 nm with SM/PM fiber coupled, Model: ADR-1805, Pegasus Shanghai Optical System Co. Ltd., Shanghai, output wavelength 405 ± 5 nm and FWHM of 2 nm) a fiber optics miniature spectrometer (HR 2000+, Ocean Optics, Inc., FL, USA with a resolution of 0.035 nm, entrance slit 50 μm, integration time 1ms to 65s, and spectral response range 200–1100 nm), fibers (SMA 905 to single-strand optical fiber of 0.22 NA), a USB port, and a laptop were used. The optics of the device is explained by the ray diagram as depicted in Fig. 1b. It can be viewed that laser light felt on the beam splitter (UVFS Plate Beam splitter, Model: BSS10R, 25 × 36 nm 30:70 (R: T), Newton, NJ, USA) through a collimating lens (74-series Collimating Lenses, 200–2000 nm Ocean Optics, FL, USA) and splits into two components i.e., one along the direction of the incident ray and the other at a right angle. Light at the right angle falls on the oral cavity tissue through a focusing lens (Achromatic Doublets, AR Coated: 400–700 nm, Thorlabs, Newton, NJ, USA, f = 2.1 cm). At the lower part of the probe, a disposable cap made of Teflon material (Virgin PTFE, HINDUSTAN NYLONS, Maharashtra, India) was placed in this manner that focused light drop at the end of the tip so that maximum fluorescence signal could be achieved. Fluorescence signals produced by the fluorophores of the tissue are recorded by the spectrometer. A LPF and a CL are placed back into the spectrometer to eliminate specular reflection and to get collimated light respectively. For the clinical testing, we installed the device in the Hallet Hospital also known as Lala Lajpat Rai Hospital Kanpur, UP, India. Photograph of the device with the accessories is shown in Fig. 1c. While performing the measurements on patients and volunteers, laser light (λ_exc_ = 405 nm) was shined on the buccal mucosa, and fluorescence signals were recorded by the UV-visible spectrometer. These measurements were conducted in a dark room with all the lights turned off. A torch was used only to position the device on the abnormal region of the oral cavity and turned off before recording the spectra. Laser light of incident power (≈ 122 µW; on the surface of the oral cavity) and the integration time of 3s was much enough to get the fluorescence signal from all the anatomical sites of the oral cavity.

### Data collection

The probe tip, as shown in the image (Fig. 1c) was placed in the abnormal area of oral cavity. Identification of biopsy area was done with the help of doctors and the probe was positioned nearby to record spectra. Data was acquired from 79 buccal mucosal (BM) sites of 36 OSCC patients, 48 BM sites of 19 dysplastic patients, and 62 BM sites of 36 healthy volunteers by fluorescence portable device. Since the probe can scan an area of 1mm^2^, therefore two to five spectra were recorded per patients, depending on lesions size. The mean age with the SD values of the OSCC, dysplastic, and control group were 45 ± 11, 36 ± 7, and 34 ± 6 respectively. The following points were taken care of at the time of diagnosis. (i) Patients were notified in advance not to ingest any kind of food or liquor on the day of treatment. (ii) A brief description of the working of the fluorescence device and its use fullness over the biopsy was explained. (iii) Patients and volunteers were advised to clean their oral cavities. (iv) Volunteers free from disease and unhealthy habits like consuming tobacco-based products were included. (v) A consent form was collected. (vi) A questionnaire form was also given to them in which their details such as age, background of the family, lifestyle, occupation, and habits of consuming unhealthy products were questioned. After recording the spectroscopic data from OSCC and dysplastic patients, they were sent for biopsy.

### Follow-up of ethical approval

We had taken the ethical approval with the IEC communication number IITK/IEC/2015-16/2/10 to conduct this clinical work. It was provided by the committee members of IIT Kanpur and GSVM Medical College, UP, India. We also took approval from clinical trials registry India (CTRI) with registration number CTRI/2017/10/010102.

### Analysis methods (PCA, LDA, QDA, ROC)

**Fig. 2:**
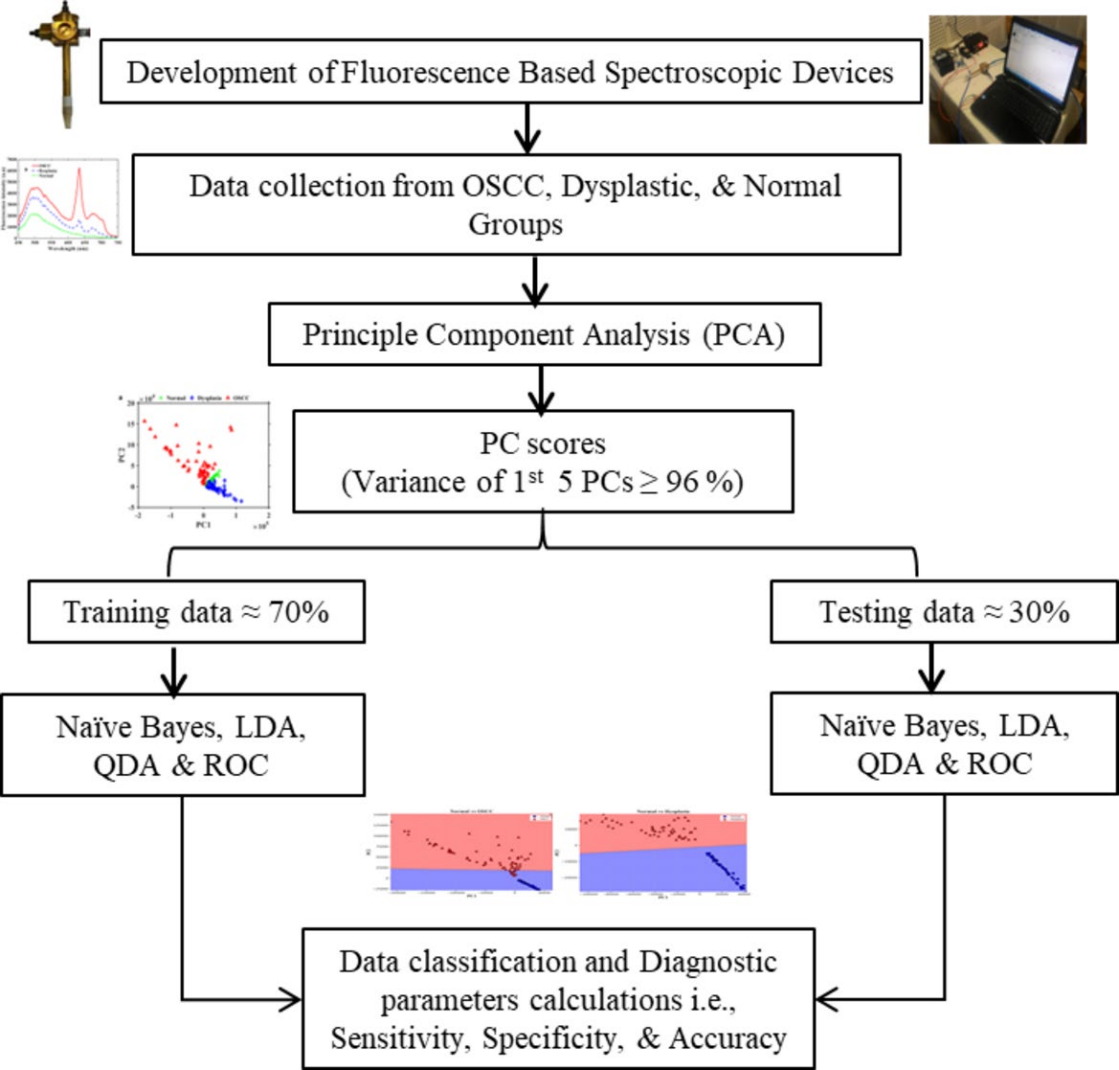
All the series of steps to classify the fluorescence data using PCA, Naïve Bayes, LDA, QDA, and ROC.

To analyze the data, we first employed principle component analysis (PCA) on the data. The purpose of employing PCA on a data set was to reduce the dimension without missing the essential features. It could be simply done by computing the eigenvectors, also called principal components. The first five eigenvalues that capture the total variance of > 96% were selected for further use. Further, Naïve Bayes classifier, linear discriminant analysis (LDA), quadratic discriminant analysis (QDA) were applied to classify among the three groups, i.e., OSCC, dysplastic, and normal. ROC was used for the evaluation of accuracy, sensitivity, specificity, and AUC values^35–38^. MATLAB software (MATLAB R2016a, Math Works, Massachusetts, USA) and Python (version 3.9) were utilized to analyze the data. All the series of steps from the development of the fluorescence device to data classification are summarized in the flow chart as depicted in Fig. 2.

## Results

**Fig. 3:**
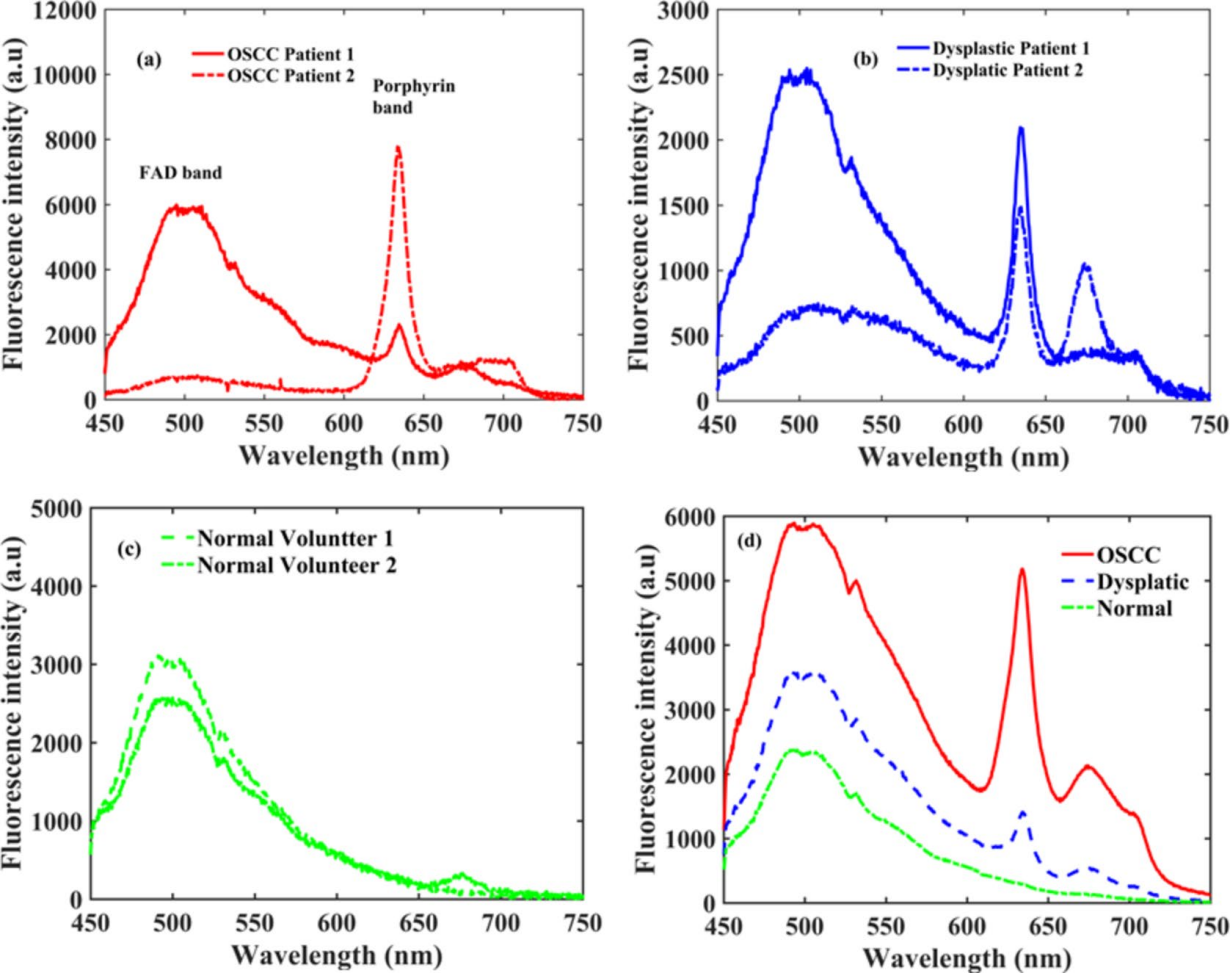
Typical fluorescence spectra of (**a**) two OSCC patients (**b**) two dysplastic patients (**c**) two normal volunteers (**d**) averaged spectra of all the patients and volunteers.

Fluorescence spectra are recorded from buccal mucosa (BM) of the oral cavity at λ_exc_ = 405 nm in the spectral range of 450 to 750 nm as displayed in Fig. 3a-d. Fluorescence emission bands of FAD (λ_emi_ = 500 nm) and porphyrin (λ_emi_ = 634, 676, 689, and 703 nm) are noticed in the spectra. Typical spectra of two OSCC and dysplastic patients and for two normal volunteers are depicted in Fig. 3a-c respectively. In the spectra, prescence of FAD band at 500 nm can be viewed in all three groups. However porphyrin bands (major at 634 nm & minor at 676, 689, and 703 nm) can be noticed in OSCC and dysplastic patients only. Fluorescence spectra of patients and volunteers comprise two dissimilar types of spectral profiles for each. For patient 1 as depicted in Fig. 3a, the fluorescence intensity of the FAD band (λ_emi_ = 500 nm) is larger than the porphyrin band (λ_emi_ = 634 nm). In patient 2, the intensity of the FAD band is lower than the porphyrin band. We also noticed the exitence of porphyrin bands in approximately 85% of OSCC patients. Along with it, in half of the OSCC patients, porphyrin bands intensities were significant prominet than the FAD bands. Two distinct spectra of dysplastic patients are depicted in Fig. 3b. Here almost in ≈ 70% patients, we observed the exitence of porphyrin bands in which on ≈ 30% patients, porphyrin bands were prominent over the FAD bands. In the spectra of the normal volunteers, FAD bands can only be viewed in both. However, a small band of porphyrin can be seen in Volunteer 2. Out of 36 volunteers, very weak bands of porphyrin at 634 nm are seen in only two volunteers and it may be due to bacterial infections in the oral cavity. An averaged spectra of all three groups is displayed in Fig. 3d. It indicate that the fluorescence intensity of the porphyrin bands for OSCC are close to the FAD bands. The intensity of the porphyrin band for dysplastic is lesser than the FAD. However, in the control group, one could not see the porphyrin band.

## Discussion

**Fig. 4:**
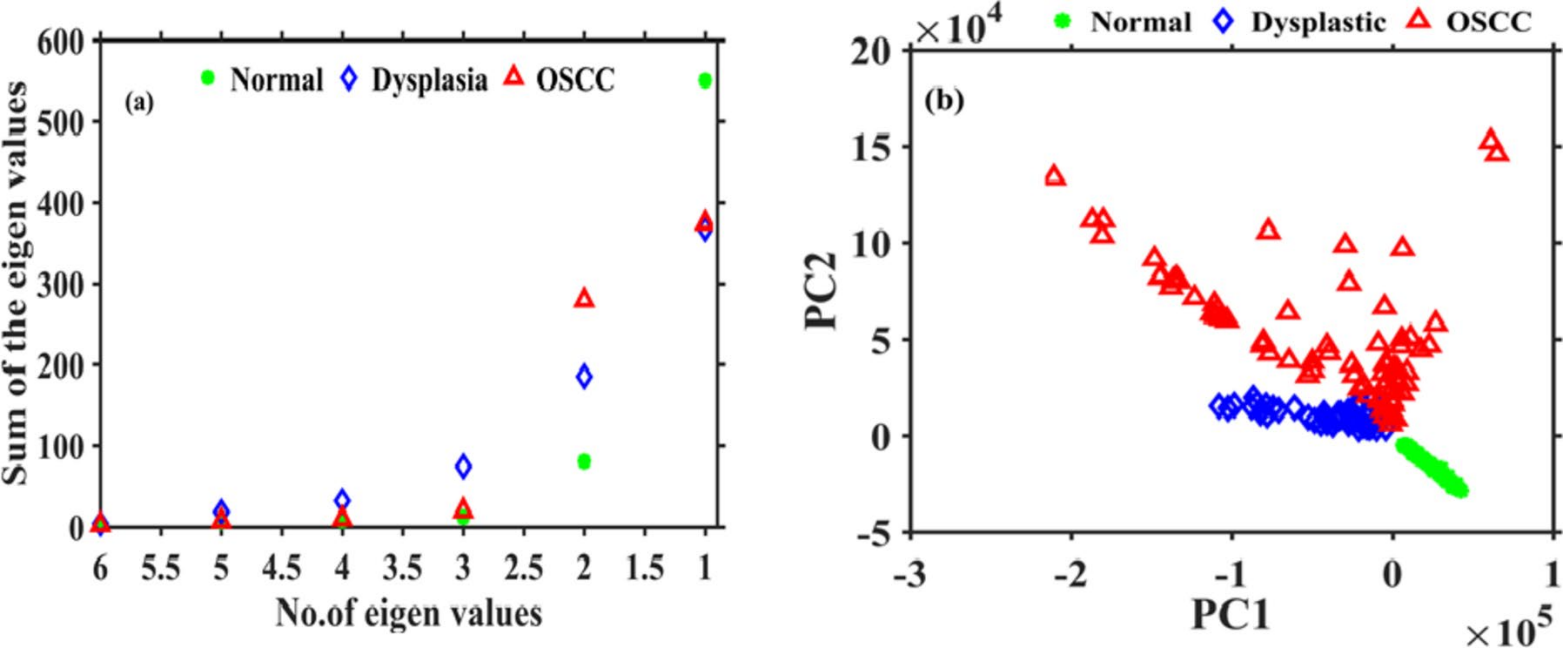
(**a**) Eigenvalue plot of first 6 PC scores (**b**) scatter data plot of first two PC scores.

The incremennt of the porphyrin bands intensity and the supression of FAD intensity values reveal that the concentration of fluropores had altered with the advanceent of oral carcinoma (**Normal → Dysplastic → OSCC**). Spectral profiles of patients (Fig. 3d) indicate that porphyrin bands will not be promient over the FAD band in all the cases. To distinguish the various stages of oral cancer, we took first the the maximum values of intensities of porphyrin (I_porphyrin_) and FAD (I_FAD_) bands from all three groups and then computed their ratio. ROC applied on the ratio values (I_Porphyrin_/I_FAD_) was not able to discern the groups with significant sensitivity and specificity values. Therefore, we concluded that analyzing the data by opting the specific biomarkers (I_porphyrin_/I_FAD_) will not be a right approach. Analyzing the data by the selection of entire spectra might be a good alternative. To improve the diagnostic efficacy, we further took the entire spectral range (450 to 750) for the data analysis. The original recorded data has a dimension of 700. Feature extraction was executed by applying the PCA and the first five PC scores consisting of a variance of more than 96% were taken for further analysis. Out of the five PC scores, the first two PCs (PC1 & PC2) consist of a total 91% percent variance. It indicates that the major information is in the first few PCs. An eigenvalue plot for the first six eigenvalues as depicted in Fig. 4a shows that all key information is available in 1st five eigenvalues. Scatter data plot for 1st two PC scores (Fig. 4b) shows that there is an overlap between OSCC and dysplastic groups. However, normal is well separated from both the groups.

**Fig. 5:**
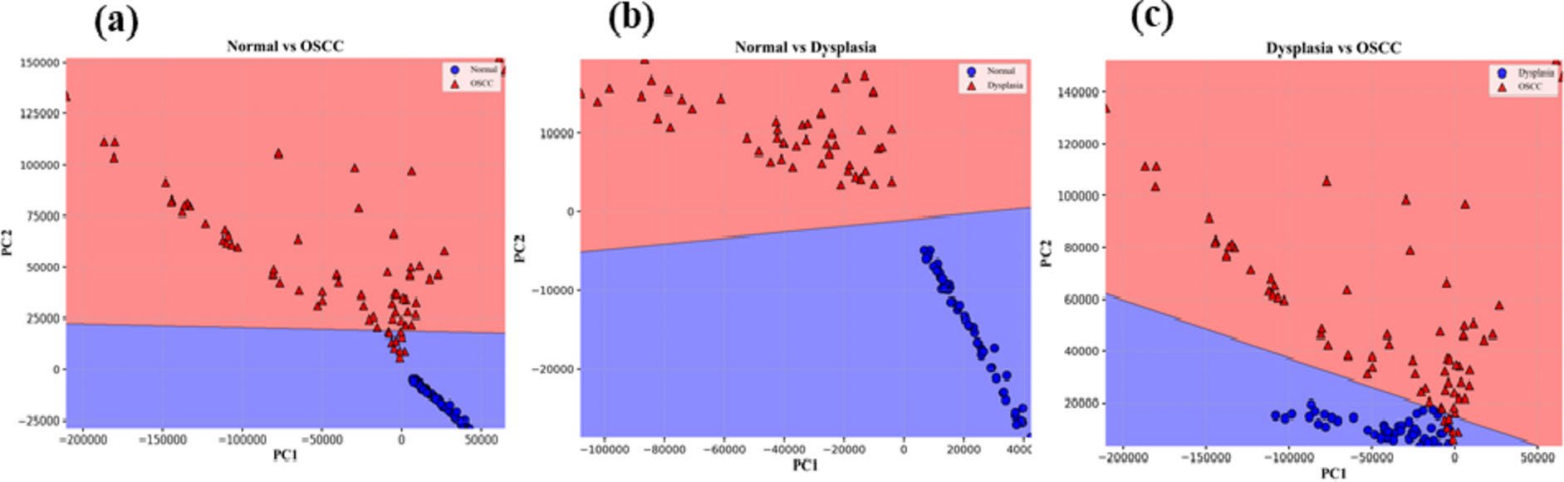
Naïve Bayes scatter plots among Normal, Dysplasia, and OSCC groups.

**Fig. 6:**
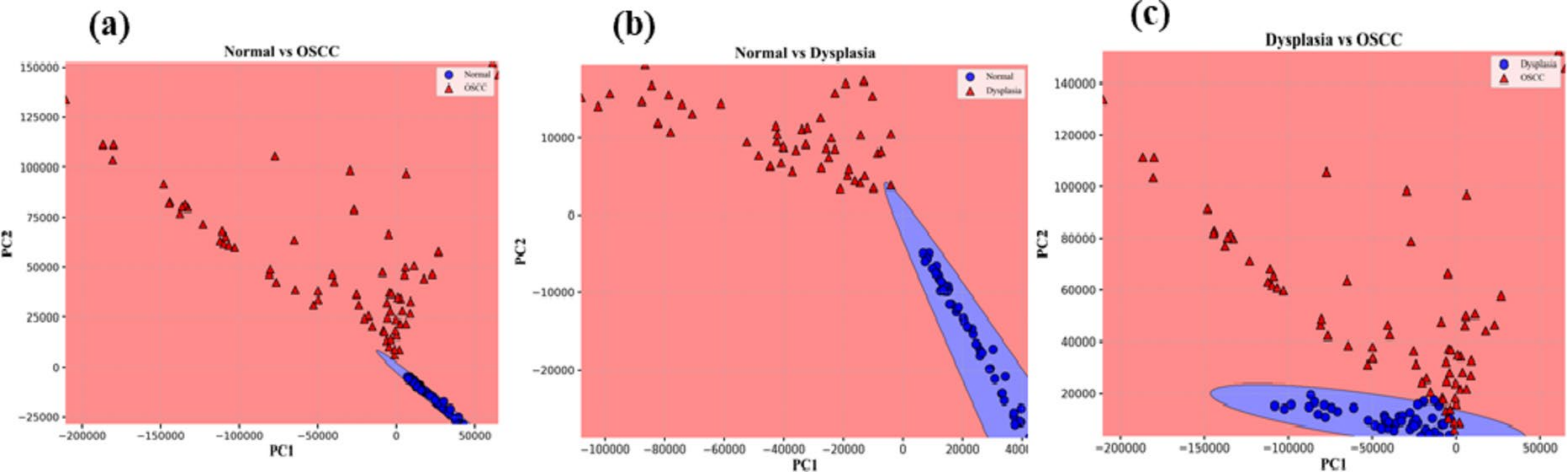
LDA scatters plots among Normal, Dysplasia, and OSCC groups.

**Fig. 7:**
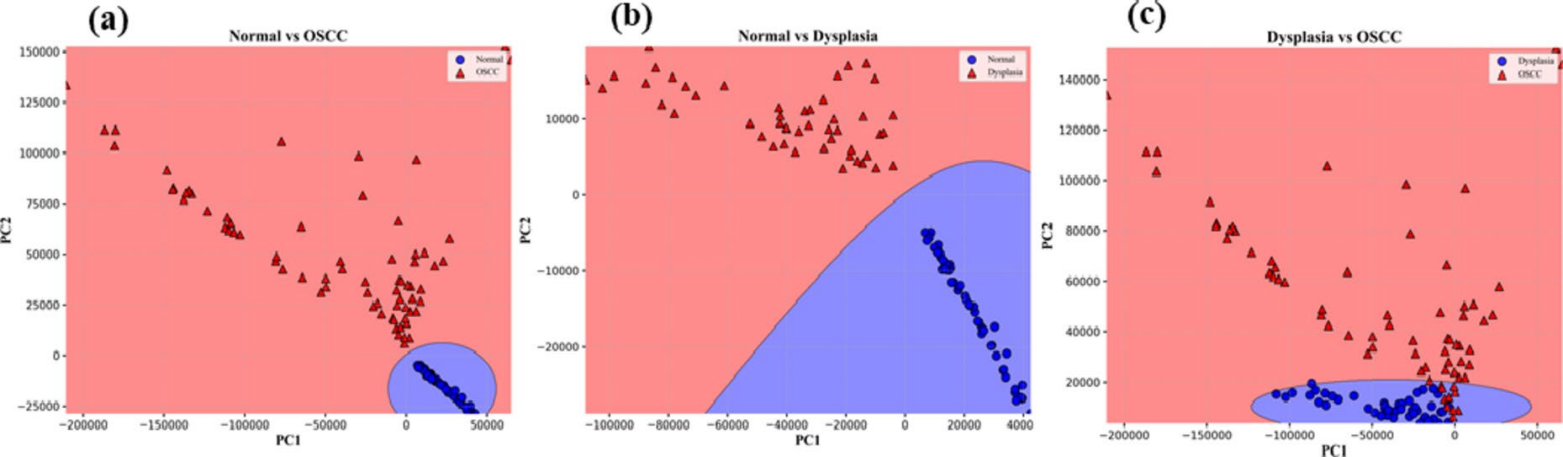
QDA scatters plots among Normal, Dysplasia, and OSCC groups.

Before applying the Naïve Bayes, LDA, and QDA classifiers on the PC scores, PC scores are segregated into two sets i.e., training and testing data sets. We took 70 and 30% data for training and testing sets respectively. Scatter data plots obtained for Naïve Bayes, LDA, and QDA applied on first two PC scores are displayed in Figs. 5a-c and 6a-c, and Fig. 7a-c respectively.

It can be seen in the above scatter plots that there are overlaps among the data points especially in between dysplasia to OSCC groups. It reflects that analyzing the data by incorporating only two PC scores had shown slightly poor diagnostic parameters. We therefore selected 5 PC scores and applied all the AI algorithms on these PC scores. The confusion matrix for training and testing data sets of all three groups obtained from Naïve Bayes, LDA, and QDA, are depicted in Table 1. In the Normal vs. OSCC groups of training data, we have TN = 40, FP = 0, FN = 1, and TP = 57 for Naïve Bayes. However, for testing data, we have TN = 22, FP = 0, FN = 0, and TP = 21. Sensitivity = TP/TP + FN, Specificity = TN/TN + FP, and accuracy = TP + TN/TP + TN + FN + FP are calculated by use of these formulas.

**Table 1.** Confusion matrix for (i) naïve Bayes (ii) LDA (iii) QDA data sets

	Normal Vs. OSCC				Normal Vs. Dysplasia				Dysplasia Vs. OSCC			
	Training		Testing		Training		Testing		Training		Testing	
(i)	Confusion Matrix for Naïve Bayes											
	40	0	22	0	40	0	18	0	31	1	15	1
	1	57	0	21	1	33	0	15	7	49	4	19
(ii)	Confusion Matrix for LDA											
	40	0	22	0	40	0	18	0	31	1	15	1
	1	57	0	21	1	33	0	15	6	50	5	18
(iii)	Confusion Matrix for QDA											
	40	0	22	0	40	0	18	0	31	1	15	1
	0	58	0	21	0	33	0	15	1	55	0	23

**Fig. 8:**
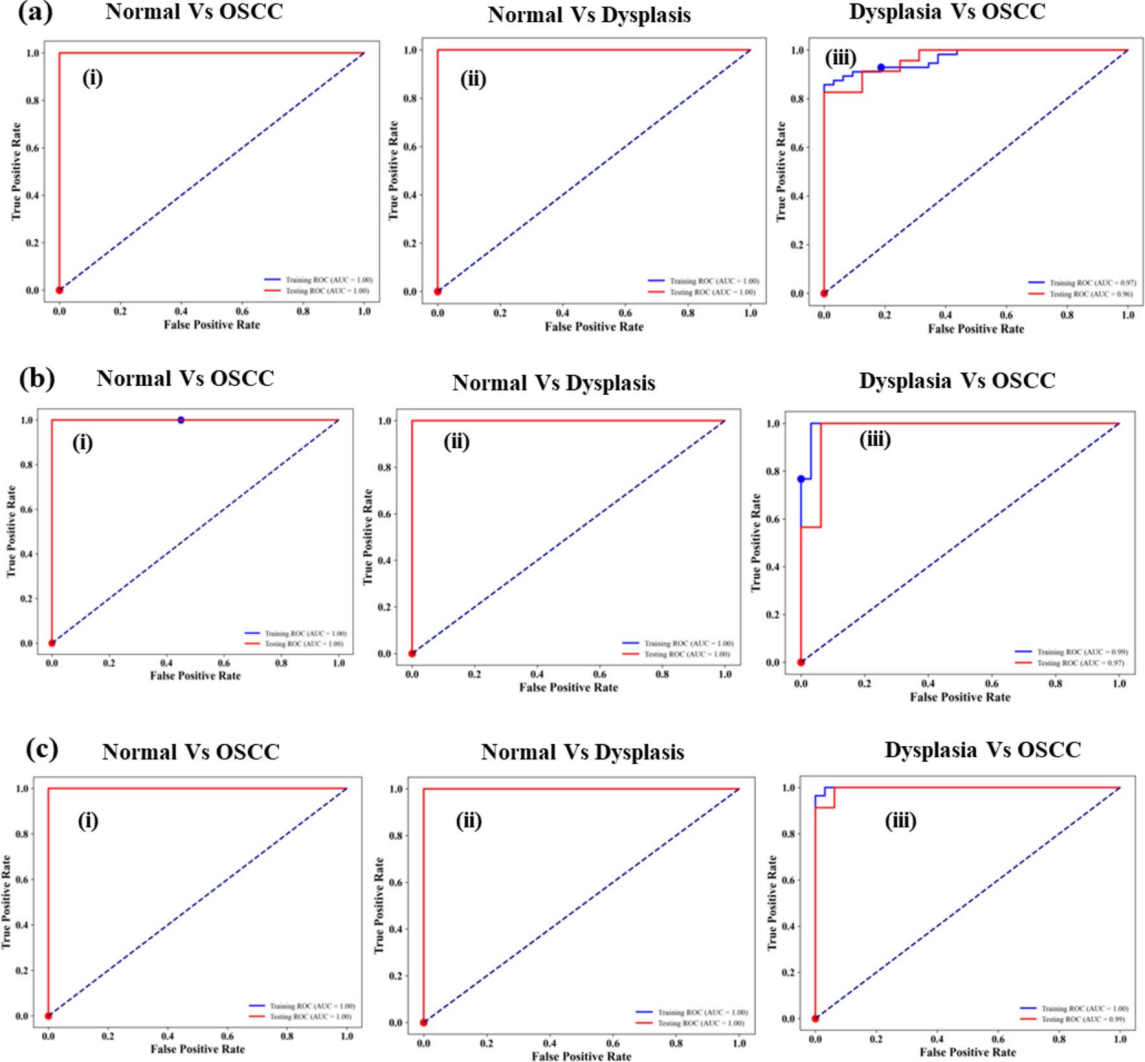
ROC curves among the groups applied on (**a**) Naïve Bayes data (**b**) LDA data (**c**) QDA data.

In the testing data of Normal vs. OSCC and Normal vs. Dysplasia, we obtained 100% sensitivities in all three classifiers. While differentiating Dysplasia from OSCC, we obtained 82.60%, 78%, and 100% sensitivity values for Naive Bayes, LDA, and QDA. It shows that QDA was able to differentiate these two groups with higher values of sensitivity. ROC applied on few random data of Naïve Bayes, LDA, and QDA had not shown a significant change in the diagnostic parameters. ROC curves generated in the process are depicted in Fig. 8a-c. AUC values are significantly high (AUC = 1) for training and testing data sets for Normal vs. OSCC and Normal vs. Dysplasia for each AI tools. However in differentiating Dysplasia Vs OSCC, it is higher for QDA (AUC = 0.99), which confirms that QDA is slightly better AI tool than the naïve Bayes and LDA.

Advanced AI methods, such as neural networks and ensemble techniques like Random Forest, have demonstrated superior performance over traditional classifiers such as Naïve Bayes, Linear Discriminant Analysis (LDA), and Quadratic Discriminant Analysis (QDA). While the latter are relatively simple and effective for linear or Gaussian-distributed data, they often struggle with complex, nonlinear relationships present in real-world datasets.

Neural networks, with their deep learning capabilities, and ensemble methods, which leverage multiple decision trees to enhance predictive accuracy, typically achieve higher performance metrics. In our study, both methods yielded exceptionally high scores, reaching 100% accuracy, sensitivity, specificity, precision, and F1-score. While this may initially suggest outstanding model performance, it is indicative of overfitting due to the small sample size. Overfitting occurs when a model learns patterns too specific to the training data, leading to poor generalization on unseen data. Thus, while neural networks and Random Forest outperform simpler methods in terms of raw performance metrics, their results must be interpreted with caution. The observed overfitting suggests the need for larger datasets, cross-validation techniques, or regularization methods to ensure a more generalizable model^39^.

## Conclusion

A clinical study was executed here to discriminate the oral mucosal lesions using the portable device and PCA-based Naïve Bayes, LDA, QDA artificial intelligence tools. With the use of the fluorescence-based device, measurements were performed on OSCC and dysplastic patients. Healthy volunteers were also included in the clinical study. In the fluorescence spectra, two prominent bands of FAD and porphyrin were noticed in the visible region near 500 nm and at 634 nm along with very few trivial bands at 676, 689, and 703 nm. Porphyrin bands were found rarely in the control group. The intensity of porphyrin bands in most of the OSCC patients was higher or comparable to FAD bands. However, in dysplastic patients, porphyrin band intensity was higher in approximate 30% cases. Classification accomplished by Naïve Bayes, LDA, and QDA tools was able to discern among the groups i.e., Normal/OSCC, Normal/Dysplasia, and Dysplasia/OSCC with the overall accuracies of 100%, 100%, 87%; 100%, 100%, 85%; and 95%, 100%, 97% respectively. Results manifest that portable device along with the PCA-based QDA tool could be a good substitute for in-vivo identification of oral mucosal lesions.

## Data Availability

The datasets used and/or analysed during the current study available from the corresponding author on reasonable request.
